# Whole Genome Sequence Analysis Using JSpecies Tool Establishes Clonal Relationships between *Listeria monocytogenes* Strains from Epidemiologically Unrelated Listeriosis Outbreaks

**DOI:** 10.1371/journal.pone.0150797

**Published:** 2016-03-07

**Authors:** Laurel S. Burall, Christopher J. Grim, Mark K. Mammel, Atin R. Datta

**Affiliations:** Center for Food Safety and Applied Nutrition, Food and Drug Administration, Laurel, Maryland, United States of America; University of Illinois at Chicago College of Medicine, UNITED STATES

## Abstract

In an effort to build a comprehensive genomic approach to food safety challenges, the FDA has implemented a whole genome sequencing effort, GenomeTrakr, which involves the sequencing and analysis of genomes of foodborne pathogens. As a part of this effort, we routinely sequence whole genomes of *Listeria monocytogenes* (Lm) isolates associated with human listeriosis outbreaks, as well as those isolated through other sources. To rapidly establish genetic relatedness of these genomes, we evaluated tetranucleotide frequency analysis via the JSpecies program to provide a cursory analysis of strain relatedness. The JSpecies tetranucleotide (tetra) analysis plots standardized (z-score) tetramer word frequencies of two strains against each other and uses linear regression analysis to determine similarity (r^2^). This tool was able to validate the close relationships between outbreak related strains from four different outbreaks. Included in this study was the analysis of Lm strains isolated during the recent caramel apple outbreak and stone fruit incident in 2014. We identified that many of the isolates from these two outbreaks shared a common 4b variant (4bV) serotype, also designated as IVb-v1, using a qPCR protocol developed in our laboratory. The 4bV serotype is characterized by the presence of a 6.3 Kb DNA segment normally found in serotype 1/2a, 3a, 1/2c and 3c strains but not in serotype 4b or 1/2b strains. We decided to compare these strains at a genomic level using the JSpecies Tetra tool. Specifically, we compared several 4bV and 4b isolates and identified a high level of similarity between the stone fruit and apple 4bV strains, but not the 4b strains co-identified in the caramel apple outbreak or other 4b or 4bV strains in our collection. This finding was further substantiated by a SNP-based analysis. Additionally, we were able to identify close relatedness between isolates from clinical cases from 1993–1994 and a single case from 2011 as well as links between two isolates from over 30 years ago. The identification of these potential links shows that JSpecies Tetra analysis can be a useful tool in rapidly assessing genetic relatedness of Lm isolates during outbreak investigations and for comparing historical isolates. Our analyses led to the identification of a highly related clonal group involved in two separate outbreaks, stone fruit and caramel apple, and suggests the possibility of a new genotype that may be better adapted for certain foods and/or environment.

## Introduction

*Listeria monocytogenes* is the causative agent of listeriosis, an invasive disease associated with high hospitalization and mortality rates. Human listeriosis is typically caused by the ingestion of ready-to eat (RTE) foods contaminated with *L*. *monocytogenes*. Historically, foods typically associated with invasive listeriosis have included deli meat and soft cheeses; however, there has been a recent expansion to include ice cream and fresh produce, like cantaloupes, celery, and apples [1–3]. RTE foods pose higher risk for listeriosis as they are ingested without any further processing, such as cooking, that would kill *L*. *monocytogenes*. Many of these foods use refrigeration, among other methods, to restrict bacterial growth during their shelf-life. While these standard practices work well for most bacteria, they are not adequate for *Listeria* control as the organism is capable of growth at refrigeration temperature and is often tolerant to freezing temperature, high salt and low pH.

While the proactive approach to illness prevention is restricting human exposure to contaminated foods, during ongoing outbreak epidemiological investigations, timely removal of the suspected contaminated foods is essential to limit the size of the outbreak. Historically, identification of the suspected foods has been done via patient interviews (exposure history) and genome based comparisons, such as Pulsed Field Gel Electrophoresis (PFGE), to match clinical isolates with food and/or environmental isolates. These processes are lengthy and resource intensive and certain PFGE profiles are more common than others due to PFGE’s comparatively low discriminatory power, when compared to WGS, whole genome MLST (wgMLST), and core genome MLST (cgMLST) among other techniques [4], which hinders the linkage of cases that cannot be connected via traditional epidemiological approaches. In the case of human listeriosis outbreaks, epidemiological links can be obscured by low overall incidence and a wide range of incubation times (3–90 days) before the onset of illness. Coupled with these drawbacks, cases with an unknown link can go unidentified if they are infrequent enough to appear as sporadic cases. This possibility is even more likely after taking into account limitations in patient memory after the protracted incubation time or the inability to conduct patient interviews in some cases.

In order to improve our resolution of genetic relatedness, the CDC and U.S. FDA have started utilizing whole genome sequencing (WGS)to compare patient, food and environmental isolates[5, 6]. This type of approach has already assisted in linking cases associated with a specific vehicle and source [7–10]. Additionally, other groups have been able to use these approaches to identify linkages between historical isolates, aiding in source attribution [8, 11, 12]. Analysis of WGS data, however, can be difficult due to the intensive computational needs and bioinformatics skills for genomic comparisons and determining a threshold to establish relatedness. Different computational tools can also lead to some variation in the interpretation of the same data. While the overall results will likely allow similar conclusions, certain differences can be critical. Considerations such as whether to use a reference-based approach and which reference sequence to use [13] as well as differences in software algorithms can lead to different error rates, and variability leading to different interpretations [14–16]. Software algorithms may be optimized based to different assumptions either in the code or in settings chosen by the user. While in a broad analysis these differences may be unimportant, evaluation of more closely related strains could be shifted by slight differences in called SNPs. Also, many other methods can have a false high discovery rate of SNPs [16]. Understanding of these issues can make it difficult for a user to choose the right parameters when doing their analyses.

We evaluated the tetranucleotide usage pattern analysis tool, JSpecies Tetra [17], initially as a quick quality assurance/control tool for WGS and then as an initial assessment of strain relatedness, identifying isolates and potential clusters for further higher resolution comparisons. This tool is capable of rapidly analyzing several genomes on a standard personal computer (PC) via a user-friendly graphical user interface (GUI) and provides data output that is easily interpreted and communicated with a broad cross-section of the scientific and regulatory community. Further comparisons, such as average nucleotide identity (ANI), core-genome or whole genome MLST, and high quality single nucleotide polymorphisms (SNP) comparisons, can then be done to verify any relationships of interest. Using this tool coupled with sero-grouping via qPCR [18] to further streamline the process, we were able to verify previously established relationships among *Listeria* isolates, and discovered new ones including stone fruit and caramel apple outbreaks [3, 19] that may prove of use in future efforts. While addition of a qPCR sero-grouping method may seem out of date, we have found that the use of this information helps us better select comparison genomes given the distinct genomic separation of the various *L*. *monocytogenes* serotypes [20]. We can use this sero-group information to focus our comparisons on a somewhat broad group, blind to PFGE pattern similarities that can be obscured by phage variations, while still narrowing it to a dataset that a normal workstation can process. Additionally, this qPCR can be completed before a sequence run is completed or even started, providing immediate guidance to a front line analyst as to which group to compare the new data with when performing the initial JSpecies comparison. The use of the JSpecies tool will enhance our ability to quickly identify isolates with a high degree of relatedness during outbreak investigations, improving our response during attribution studies and in detecting incidents with any potential links to historic or otherwise epidemiologically unlinked *Listeria* isolates.

## Materials and Methods

### Strains, media and reagents

Strains used in this study are listed in S1 Table and Table 1. Strains examined in this study were from our lab collection, as well as a few well sequenced reference genomes from NCBI. Strains from our collection were included in this study if they were serotype 4b and had already been sequenced as part of active outbreak investigations or the GenomeTrakr project with SRA files publicly available for analysis [5] or during outbreak follow-up investigations, as with the stone fruit isolates. Strains sequenced were randomly chosen from isolates obtained during various isolation efforts, whether by our lab or the originating lab. Cultures were stored at -80^0^ C in presence of 20% glycerol and routinely grown at 37^0^ C using brain heart infusion (BHI) (Sigma-Aldrich, USA) agar or broth.

**Table 1:** Strains analyzed in the various phylogenetic comparisons highlighted in this paper.

Strain	Accession #	Alternate Designation	Source Type	Originating Lab^a^	Country^c^	Isolation Year	Serotype
**CLIP80459**	FM242711.1[21]		cheese	n/a^b^			4b
**F2365**	AE017262.2[22]		patient	n/a			4b
**H7858**	AADR00000000[22]		meat	n/a			4b
**HPB2262**	AATL00000000[23]		patient	n/a			4b
**J1-220**	CP006047.2[24]		patient	n/a			4b
**J1776**	CP006598		patient	n/a			4b
**J1816**	CP006046.2[24]		environmental	n/a			4b
**J1817**	CP006599		environmental	n/a			4b
**J1926**	CP006600		meat	n/a			4b
**L312**	FR733642.2		cheese	n/a			4b
**LL195**	HF558398.1		Patient	n/a			4b
**ScottA**	CM001159.1[25]		patient	n/a	USA	1983	4b
**LS41**	LNOC00000000	CEB 1883	patient	Pasteur Institute		pre-1986	4bde
**LS42**	LNNL00000000	CEB 1890	patient	Pasteur Institute		pre-1986	4bde
**LS47**	LNOD00000000	CEB 2015	environmental	Pasteur Institute		pre-1986	4bde
**LS50**	LNOE00000000	CEB 2776	animal	Pasteur Institute		pre-1986	4bde
**LS114**	LNNY00000000	SLCC 4527	patient		Germany	1975	4b
**LS267**	LNNM00000000	BE4-79	patient	TX DOH	USA	1994	4bde
**LS268**	LNNN00000000	BE4-80	patient	TX DOH	USA	1994	4bde
**LS269**	LNNO00000000	BE4-81	patient	TX DOH	USA	1994	4bde
**LS270**	LNNP00000000	BE4-148	patient	TX DOH	USA	1994	4bde
**LS271**	LNNQ00000000	BE4-288	patient	TX DOH	USA	1994	4bde
**LS272**	LNNR00000000	BE4-769	patient	TX DOH	USA	1994	4bde
**LS275**	LNNS00000000	BR4-1259	patient	TX DOH	USA	1994	4bde
**LS276**	LNNT00000000	BR4-1292	patient	TX DOH	USA	1994	4bde
**LS542**	AVQQ01	501928	environmental	WEAC	USA	2010	4bV
**LS642**	AVQM01	10M – 127	clinical		Australia		4bV
**LS643**	AVQN01	10M – 130	clinical		Australia		4bV
**LS644**	AVQO01	10M – 138A	clinical		Australia		4bV
**LS645**	AVQP01	10M – 198	clinical		Australia		4bV
**LS651**	LNNU00000000	HUM 2011015611	patient	Denver HMC	USA	2011	4b
**LS988**	LNNV00000000	869223-5c1	yellow nectarine	OARSA	USA	2014	4bV
**LS990**	LNNW00000000	869224-1a2	white peach	OARSA	USA	2014	4bV
**LS993**	LNNX00000000	869224-2b1	white peach	OARSA	USA	2014	4bV
**LS996**	LNNZ00000000	869224-2c2	white peach	OARSA	USA	2014	4bV
**LS997**	LNOA00000000	869224-3a1	white peach	OARSA	USA	2014	4bV
**LS1000**	LNOB00000000	869224-3b2	white peach	OARSA	USA	2014	4bV
LS1012*	LNPM00000000	824917–3	granny smith apple	Den-DO	USA	2015	4b
LS1013*	LNPN00000000	824917–4	granny smith apple	Den-DO	USA	2015	4b
LS1014*	LNPO00000000	824917–5	granny smith apple	Den-DO	USA	2015	4bV
LS1015*	LNPP00000000	824917–6	granny smith apple	Den-DO	USA	2015	4b
LS1016*	LNPQ00000000	824917–7	granny smith apple	Den-DO	USA	2015	4b
LS1017*	LNSR00000000	824917–10	granny smith apple	Den-DO	USA	2015	4bV
LS1023*	LNSL00000000		patient	CDC	USA	2014	4b
LS1024*	LNSM00000000		patient	CDC	USA	2014	4bV
LS1064*	LNSN00000000	Lm 2014 L-6268	patient	CDC	USA	2014	4bV
LS1065*	LNSO00000000	Lm 2014 L-6561	patient	CDC	USA	2014	4bV
LS1066*	LNSP00000000	Lm 2014 L-6390	patient	CDC	USA	2014	4bV
LS1067*	LNSQ00000000	Lm 2014 L-6470	patient	CDC	USA	2014	4bV

^a^Originating Lab refers to the group that provided the isolates to our collection.

^b^n/a, Genomes were accessed through NCBI and the strain was not used from our collection.

^c^Country, refers to the original country of origin of the isolate.

*Indicates strains sequenced at their originating labs prior to inclusion on our collection.

### Sero-grouping of isolates

A qPCR method for sero-grouping has been developed based on a modification of a previously published conventional PCR method [18, 26]. The qPCR primers and probes are shown in Table 2. The reaction was performed using Quantifast Multiplex PCR kit without ROX (Qiagen, Germantown, MD) in two separate multiplex reactions. The first reaction verifies that the isolate is *Listeria monocytogenes* via *Listeria* genus and *L*. *monocytogenes* species specific targets. The second reaction determines which serogroup the isolate belongs to, if any (Table 3). Results of the sero-grouping analysis are reported in Table 1.

**Table 2:** qPCR Primers and Probes.

Name	Sequence	5’ Fluorescence Label	3’ Quencher
**IAC-PCF610**	TTACAACGGGAGAAGACAATGCCACCA	CAL Fluor Red 610	BHQ-2
**Lall-PFAM**	ATGTCATGGAATAAT	FAM	MGB-NFQ
**Hly3-PTET**	TGCACTGGTTTAGCTTGGGAATGGT	TET	BHQ-2
**lmo0737-Q670**	TTTGCAAGTCAGGGTCTTGTCCGA	Quasar 670	BHQ-2
**lmo1118-TET**	AGGCGTATACACTCAGGAGAAGATAAAGGT	TET	BHQ-1
**ORF2110-Q705**	AGTATGACTTCGGGCACAGTTGGC	Quasar 705	BHQ-2
**ORF2819-FAM**	TGGCAGTTTCCAGGACTTCACTTGT	FAM	BHQ-1
**IAC_F**	GGCGCGCCTAACACATCT	n/a	n/a
**IAC_R**	TGGAAGCAATGCCAAATGTGTA	n/a	n/a
**qhly3_F**	GCTCATTTCACATCGTCCATCTA	n/a	n/a
**qhly3_R**	CCGGTCATCAATTACCGTTCTC	n/a	n/a
**qiap_F**	GTTAAAAGCGGYGAYACWATTTGG	n/a	n/a
**qiap_R**	TTTGACCYACATAAATAGAAGAAGAAGATAA	n/a	n/a
**qlmo0737_F**	AGATGAACGGCAGAGACTTAAA	n/a	n/a
**qlmo0737_R**	CCGATCCGAATGCTGCTAATA	n/a	n/a
**qlmo1118_F**	TGCTTAATAACAGATGAAGAGGATG	n/a	n/a
**qlmo1118_R**	CTTGTTCCTTAGTATTCCAGGATTT	n/a	n/a
**qORF2110_F**	CAGAATACGGCATCCCTGATAA	n/a	n/a
**qORF2110_R**	AGCTCCACGTCCAAAGTAAG	n/a	n/a
**qORF2819_F**	CATCACTAAAGCCTCCCATTGA	n/a	n/a
**qORF2819_R**	CCCTCCAACATATACGGAAAGAG	n/a	n/a

**Table 3:** Serogroup Pattern Identification.

	2a Complex	2b Complex	2c Complex	4b Complex	4bV
**Indicated Serotype**	1/2a, 3a	1/2b, 3b	1/2c	4b, 4d, 4e	4bV
***lmo1118***	-	-	+	-	-
***lmo0737***	+	-	+	-	+
**ORF2110**	-	-	-	+	+
**ORF2819**	-	+	-	+	+

### Genomic DNA isolation and sequencing

Genomic DNA was isolated on a QiaCube (Qiagen, Germantown, Maryland) using Qiagen’s DNeasy Blood & Tissue kit and the Gram Positive extraction protocol with the pre-lysis step incubated for 1 hour at 37°C. The DNA concentration was determined using a Qubit 2.0 fluorometer (ThermoFisher Scientific) and then diluted to 0.2ng/uL. Genomic libraries were prepared with a Nextera XT DNA sample preparation kit (Illumina, San Diego, CA). A 2x250 paired-end sequencing run was performed on an Illumina MiSeq benchtop sequencer and reads were trimmed and assembled using the CLC Genomics Workbench v7.0 (CLC Bio, Aarhus, Denmark). The assembled contigs were exported as fasta files for JSpecies analyses. Assembly files for strains involved in the detailed analyses have been submitted to NCBI (Table 1).

### Genomic analyses by JSpecies

Sequences were imported into the JSpecies workspace and a Tetra analysis was performed [17]. A subset of the isolates was also analyzed in the JSpecies workspace using the ANIb (average nucleotide identity via BLAST) analysis tool to further verify the results. The data file was exported to Microsoft Excel and sorted numerically to rank pairs by their r^2^ value for Tetra analysis or percent identity for ANI comparisons.

### Genome comparison by SNP analysis

Two versions of SNP analyses were performed on the data. The first was performed using a BLAST-based SNP analysis that first identified a core set of genes (n = 2052). This core set of *Listeria monocytogenes* genes was selected using 92 whole genome sequences (11 closed genomes and 81 shotgun sequences) that were incorporated in a BLAST database. All annotated genes of the reference strain *Listeria monocytogenes* F2365 (GenBank AE017262) were BLASTed against the database. Each gene that was determined to be present in each of the 92 genomes exactly once was retained as a core gene. A gene was considered present in a genome if it matched the genome at least 90% sequence identity over at least 90% of the gene length. As a result, 1,852 genes were selected.

To identify SNPs in genes within the test strains, the core reference genes were BLASTed against the database, and for each genome matched, the matching bases to the reference sequence were aligned in one file for each core gene. Each core gene alignment was then scanned for nucleotide variation at each position. A fasta sequence was generated for each genome sequence by concatenating all of the 23,545 variable bases from the core gene alignments. These fasta sequences were aligned using MEGA6 [27] and this alignment was used to construct a phylogenetic tree using the Neighbor-Joining method with node confidence assessed by 1000 bootstrap replicates [28].

A second SNP analysis was performed using the CFSAN, FDA SNP pipeline, run locally on a Linux Ubuntu workstation. Reference strains were selected based on serotype information so that 4bV strains were mapped against an epidemiologically unrelated, but phylogenetically close, 4bV strain, LS642, and 4b strains were mapped against F2365, an unrelated 4b strain. The source code and further description is available at https://github.com/CFSAN-Biostatistics/snp-pipeline [16]. The SNP FASTA file output was used to reconstruct phylogenetic relationships using MEGA among strains and to specifically determine SNP differences within a given cluster of strains by exporting the non-conserved SNPs to a Microsoft Excel file for analysis.

## Results

### Evaluation of the JSpecies tool

JSpecies is a species-level genome sequence comparison suite that is comprised of three different peer-reviewed bioinformatics tools [17]. The focus of this work is the Tetra tool, an alignment free tool that performs pairwise analyses of genome sequences by examining the frequency of the 256 possible nucleotide tetramers. This frequency is compared with the expected frequency based on the size of the sequence and deviation from the expected frequency provides a z-score that is used to compare two strains in a linear regression analysis. This calculation allows genomes of different sizes to be compared without the results being skewed by genome completeness, which is a key consideration when comparing draft genome sequences. Each pair within a dataset is compared via a linear regression analysis which provides an r^2^ value. In linear regression analyses, an r^2^ value of 1 indicates that the data of one strain is considered to have “predicted” perfectly the data of the other strain. This means that as the r^2^ value approaches one the strains can be considered more closely related as the results for one predict/match the results for the other. From our experience, values of greater than 0.99998 are indicative of a highly probable clonal relationship. A value of 0.99998 is indicative of slight differences, some of which are real and others that may be due to differences in the sequence reads and their assemblies. It should be noted that correlation coefficients between genomes that are sequenced on the same platform to equivalent coverage levels and assembled with the same software should more accurately reflect true genomic differences. As the r^2^ value continues to decrease, genomic divergence between the isolates becomes greater.

Given the familiarity of this type of statistical method and the interpretation of r^2^ values, the results can provide an easily communicable approximation of the measure of genetic relatedness. Additionally, the analysis can be performed rapidly on any computer with Java installed with extremely limited training of the analyst. Given the limited barrier to entry in using this tool, analysts generating new sequence data would be able to compare their suspect strains against previously acquired strains, allowing more rapid identification of clusters. The presumptive linkages could then be selected for further, more intensive analysis to verify the linkage. Additionally, the use of this tool prior to submission of sequence data to NCBI would provide a quality assurance step, preventing submissions containing contaminating sequence.

Test analyses of randomly selected genomes were performed to determine how quickly JSpecies Tetra could process unbiased datasets of varying sizes. Random selection from a pool of 203 Lm genomes (S1 Table) from various serotypes, isolation dates and sources was performed using a random number generator (http://www.mathgoodies.com/calculators/random_no_custom.html). JSpecies Tetra analyses of randomly selected ten genomes (3Mb average length) could be analyzed reliably in 67 seconds with one of the six trials taking 69 seconds. Analyses of 25 genomes could be performed in 183 to 200 seconds. We were able to analyze data sets of up to 80 genomes in 30 minutes or less. The analysis did stall periodically in larger datasets or those involving more distantly related strains; however, reinitiating the process resulted in progression through the data set rather than starting from the initial point. Various factors resulted in increased analysis time including strain variability as noted as well as the operation of other programs on the computer.

These initial analyses identified earlier confirmed relationships between epidemiologically linked strains involved in various previously investigated outbreaks, verifying its ability to detect genetic links. Also, in general, isolates known to be unrelated based on earlier analyses were similarly not linked in these works. However, two epidemiologically unlinked serotype 4b strains, LS47 and LS114, were found to be highly related in these analyses, which will be further investigated in the next section.

### JSpecies Tetra analysis identified unknown links

During routine qPCR-based serotype analysis based on an adaptation of a previously published method [26] of new isolates obtained from the 2014 stone fruit outbreak/incident [9, 19] and the 2014 caramel apple outbreak [3], both related to *L*. *monocytogenes* contamination, we found a portion of these isolates belonged to the serotype 4bV (Table 1)[29]. The 4bV isolates, also termed as IVb-v1, are serotype 4b by standard antigen-antibody based serology [30] but differed from 4b by PCR-based serotyping [18, 26, 30] due to acquisition of a 6.3kb DNA found in serotype 1/2a, 3a, 1/2c and 3c strains[29, 31]. Based on this information, a small panel of 4b and 4bV isolates was selected for comparison with each other via JSpecies Tetra analysis (data not shown). This analysis indicated a high degree of relatedness between the stone fruit incident/outbreak and caramel apple outbreak isolates. To investigate whether this linkage would persist in a larger, more diverse dataset, the test dataset was expanded to include 31 4b and 19 4bV strains (Table 1). This Tetra analysis confirmed that the 4bV isolates obtained during the stone fruits investigation and the caramel apple outbreak were highly related with r^2^ values of 0.99999 or 1 (S2 Table, Cluster 3). Additionally, this analysis identified a previously unknown genetic relatedness between isolates from several cases of human listeriosis linked to frozen vegetables in the state of Texas, USA that spanned December 1993 to January 1994 and an isolate from a clinical case in Colorado, USA, from 2011 with no identified source (S2 Table, Cluster 1). This analysis also verified the previously mentioned link between LS47 and LS114 (S2 Table, Cluster 2), two isolates, one clinical and one environmental, from Europe dating back approximately 30 years.

We then decided to analyze these strains and a subset of unrelated strains via JSpecies ANIb (average nucleotide identity by BLAST). Analysis of the two strains in cluster 2 (LS47 and LS114) (S2 Table) showed 99.94% identity via ANIb analysis, indicating that the strains show some divergence perhaps due to genetic drift due to temporal factors and the disparate isolation sources (silage and a human patient). Alternatively, this could be due to genetic change during lab passage and storage over the following ~30 years. The strains indicated in cluster 1 showed 99.9–100% identity by ANIb, providing further support that there is a potential link between the 1994 isolates and the 2011 patient isolate. If this information had been available in 2011, it likely would have provided valuable guidance to investigators by suggesting a possible link with the 1994 cases and associated food products. The patient could have then been queried to determine if there was an exposure history for those products.

In 2014, FDA reported a voluntary recall by a packing company in California of whole white and yellow peaches, white and yellow nectarines, plums and pluots due to the potential of the products being contaminated with *Listeria monocytogenes*. Later investigations by CDC revealed that these products could be associated with one or more cases of human listeriosis [9]. Investigations by FDA at the time of the recall resulted in *L*. *monocytogenes* isolates from several of these fruits [19]. During the latter part of 2014 and early 2015, CDC reported a multistate listeriosis outbreak involving pre-packed caramel apples made from Granny Smith and Gala apples from a packing company in California [3]. Out of 35 illnesses, 11 were pregnancy associated and three invasive illnesses (meningitis) occurred among otherwise healthy children aged 5–15 years. *L*. *monocytogenes* isolates from the environment and apples from the packing facility were closely matched with the outbreak strains. Although these outbreaks/incidents occurred during 2014 and both were associated with fruits processed in California, no link between these outbreaks was ever identified. The results of the JSpecies analysis of isolates from these two outbreaks (S2 Table, Cluster 3) clearly show a very close genetic linkage among the recent 4bV isolates. The JSpecies ANIb tool further verified this relationship showing 99.9–100% identity between a subset of the 4bV isolates obtained from stone fruits and Granny Smith apples in both the initial and reciprocal comparisons. Importantly, comparisons with five unrelated 4bV strains identified no significant link either by JSpecies Tetra or ANIb analysis (S2 Table & Table 4).

**Table 4:** JSpecies ANIb of a subset of the 4bV Strains again shows a higher level of relatedness between the apple and stone fruit linked isolates and 4bV strains from unrelated sources.

	LS997	LS1017	LS1065	LS1014	LS1064	LS996	LS1024	LS642	LS644	LS542
**LS997**	---	99.99	99.98	99.99	99.99	99.98	99.98	99.56	99.56	99.52
**LS1017**	99.99	---	99.98	99.99	99.99	99.99	100	99.57	99.56	99.53
**LS1065**	99.99	99.99	---	99.99	99.99	99.98	99.99	99.58	99.56	99.53
**LS1014**	99.99	100	99.99	---	99.99	99.98	99.99	99.56	99.55	99.53
**LS1064**	99.99	99.99	99.97	99.99	---	99.99	99.99	99.56	99.56	99.52
**LS996**	99.99	99.99	99.99	99.99	99.99	---	99.99	99.57	99.56	99.53
**LS1024**	99.98	99.99	99.98	99.98	99.98	99.99	---	99.55	99.56	99.49
**LS642**	99.56	99.56	99.56	99.57	99.57	99.56	99.57	---	99.54	99.5
**LS644**	99.55	99.55	99.54	99.55	99.55	99.54	99.55	99.57	---	99.56
**LS542**	99.47	99.47	99.45	99.47	99.47	99.47	99.46	99.49	99.52	---

**Fig 1:**
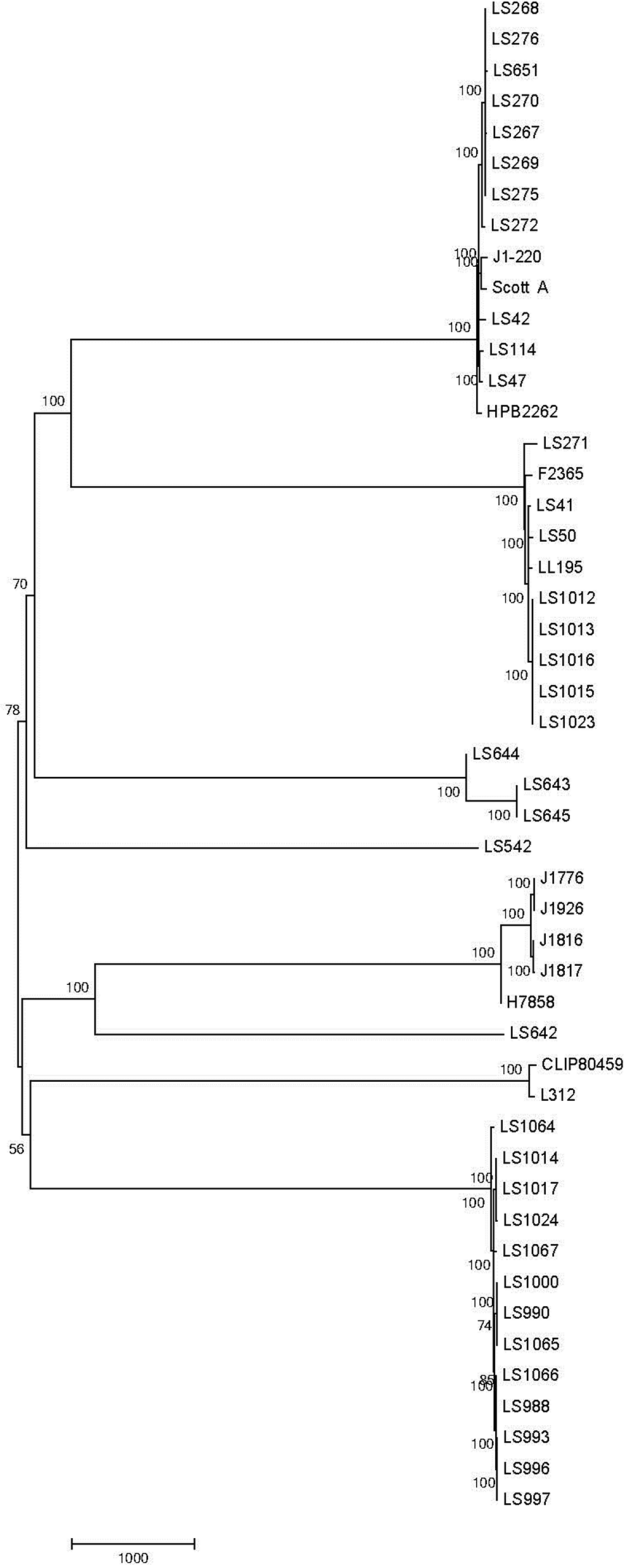
Evolutionary relationships of taxa. The evolutionary history was inferred using the Neighbor-Joining method based on the data from the BLAST-SNP analysis [22]. The optimal tree with the sum of branch length = 30986.27311707is shown. The percentage of replicate trees in which the associated taxa clustered together in the bootstrap test (1000 replicates) is shown next to the branches [32]. The tree is drawn to scale, with branch lengths in the same units as those of the evolutionary distances used to infer the phylogenetic tree. The evolutionary distances were computed using the number of differences method [33] and are in the units of the number of base differences per site. The analysis involved 49 nucleotide sequences of 4b and 4bV strains indicated in Table 1. Codon positions included were 1st+2nd+3rd+Noncoding. All ambiguous positions were removed for each sequence pair. There were a total of 23545 positions in the final dataset. Evolutionary analyses were conducted in MEGA6 [27]. The strains highlighted in Table 1 are similarly noted here with cluster 1 in purple, cluster 2 in green, and cluster 3 in blue.

**Fig 2:**
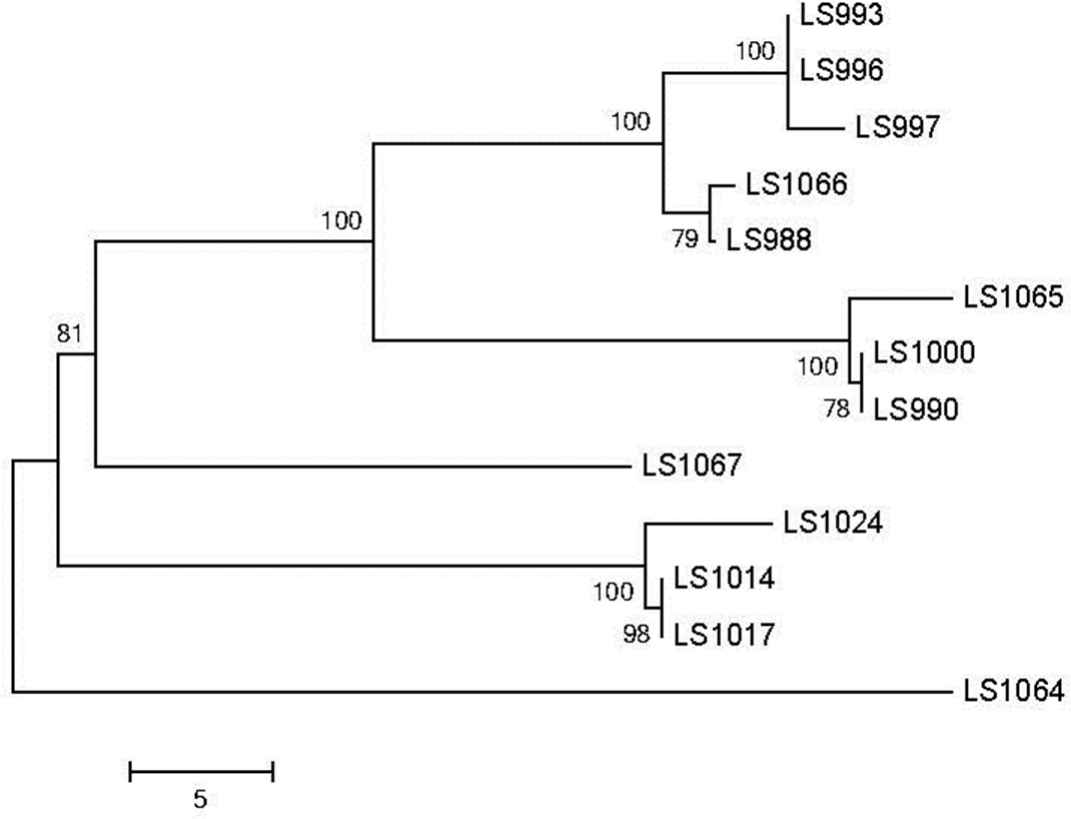
An optimal tree focusing on cluster 3 containing the stone fruit and apple 4bV isolates. The tree was generated as in Fig 1 with a sum of branch length = 129.46875000 is shown. The percentage of replicate trees in which the associated taxa clustered together in the bootstrap test (1000 replicates) are shown next to the branches [32]. The tree is drawn to scale, with branch lengths in the same units as those of the evolutionary distances used to infer the phylogenetic tree. The evolutionary distances were computed using the number of differences method [33] and are in the units of the number of base differences per sequence. The analysis involved 13 nucleotide sequences. Codon positions included were 1st+2nd+3rd+Noncoding. All ambiguous positions were removed for each sequence pair. There were a total of 23545 positions in the final dataset. Evolutionary analyses were conducted in MEGA6.

To corroborate the results from the JSpecies Tetra and ANIb analyses, a core genome SNP-based analysis was performed on the same panel of strains (Table 1). As part of this analysis, 14 genomes sequenced at other labs were also included in this dataset as they provided more complete genomic sequences and annotation, compared to the draft assemblies in this work, enabling better identification of the core genome. The BLAST-based core genome SNP comparison also showed similar links between the previously discussed strains (Fig 1). As seen with the ANIb analysis, LS47 and LS114 showed some differentiation from each other. The SNP-based tree also shows no noticeable divergence between the 1994 and 2011 isolates within the diverse dataset. A more focused comparison of the fruit 4bV strains indicates a slight divergence between the stone fruit and apple isolates (Fig 2). The outliers noted in the CDC report, which are referred to as LS1064 and LS1067 in this study, again fall outside the other clusters observed in this tree. However, the data still support a high likelihood of a recent common source for all of these isolates that warrants further consideration.

**Fig 3:**
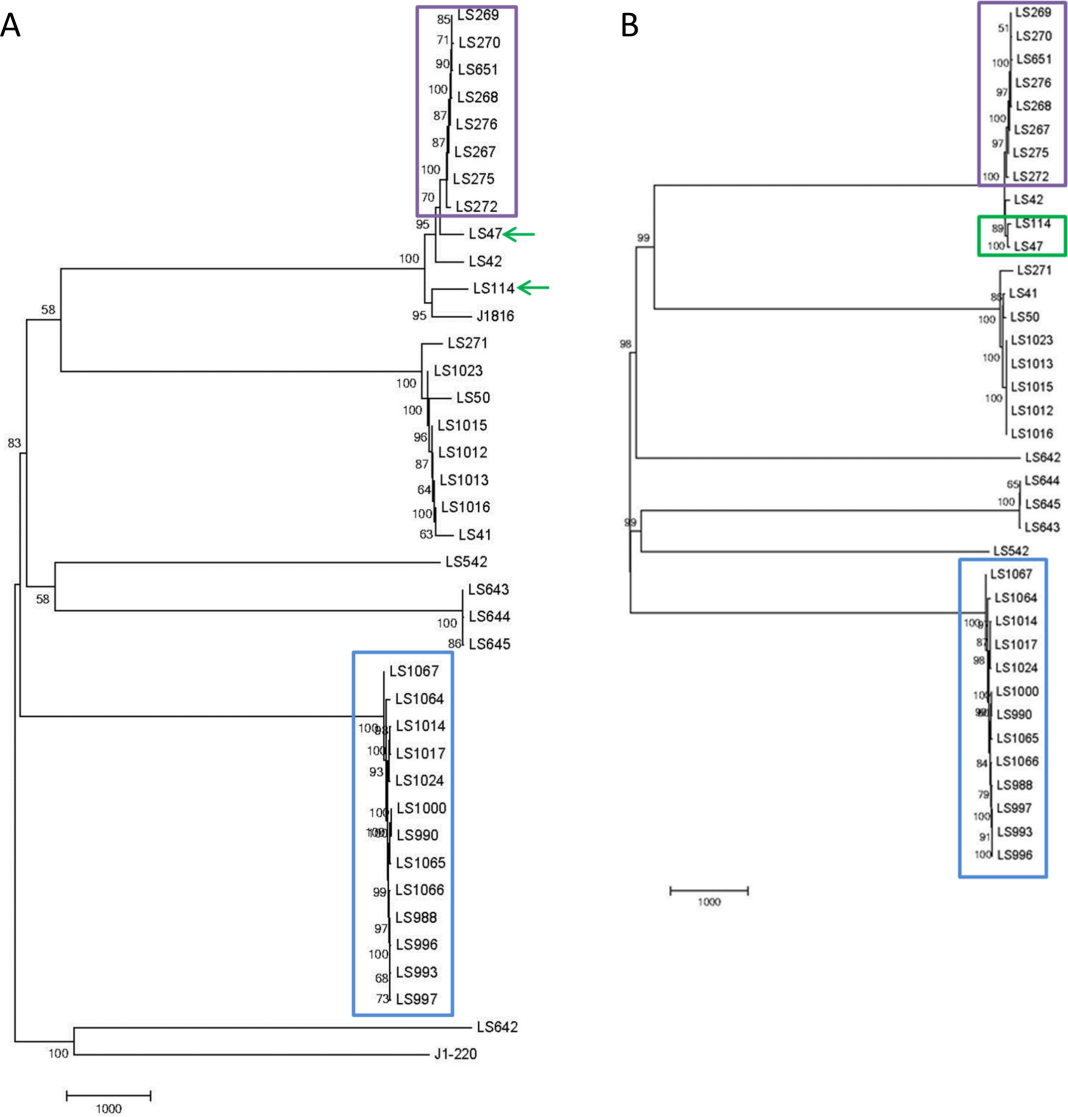
Neighbor-Joining trees based on the CFSAN SNP pipeline results. A) This tree shows the evolutionary relationships between the 4b and 4bV strains, using LS642 as the reference strain. B) This tree examines 4b and 4bV strains, using F2365 as the reference strain. Clusters 1, 2 and 3 are highlighted in purple, green and blue, respectively, in both. The use of arrows used in 3a show the alteration that can occur using a more distantly related reference strain.

Analysis of the strains was also conducted using the CFSAN SNP pipeline [16] to further validate our observations. In this case, careful consideration of a closely related reference strain was required. Given that the 4bV strains have a unique 6.3 kb island, we decided to use LS642 [29, 34], a clinical 4bV isolate from an Australian source as the reference strain for comparing the strains in cluster 3 (Fig 3A) and F2365, a serotype 4b isolate from the 1985 cheese outbreak for analysis of clusters 1 and 2 (Fig 3B) [22, 35]. As with prior analyses, we again see the strains in cluster 3 forming a clade with each other and separated from the other 4bV strains available for comparison (Fig 3A). We defined SNPs as stone fruit (SF) specific if they were present in all ten SF isolates included in this analysis and none of the apple isolates and SF biased if they were present in at least seven of the ten analyzed SF isolates and no more than one of the four apple isolates. This resulted in 22 SF specific SNPs and 25 SF biased SNPs. The converse analysis for apple specific and apple biased SNPs identified no apple specific SNPs and two apple biased SNPs, relative to the LS642 sequence. However, it was interesting to observe that in each instance of a SF specific SNP, the apple strains had the reference strain(LS642) sequence conserved at that site in 75% of the isolates with LS1014 consistently diverging (S3 Table). We also found that in all but ten of the SF biased SNPs the apple strains again had maintained the reference strain sequence. Overall, this data supports the observation that the isolates from the apple and stone fruit incidents are highly related, though not identical.

We also examined the apple associated serotype 4b strains; however, we used F2365 as the reference genome as this genome represented a closer relative than a 4bV isolate. Using this analysis, we found 11 SNPs relative to F2365, with seven of them confined to one isolate (FDA00008714). We also examined the SNP differences between LS47 and LS114 in Cluster 2 and found 126 SNPs between the two strains. The results underscore the critical need for selection of a proper reference genome as when LS642 was used as the reference strain, we found over 1000 SNPs between these two strains. However, based on the comparison using F2365 as the reference, we can conclude that the two strains are remarkably related, especially when you consider that they were originally isolated in the 1970s and have undergone an unknown number of passages, media types and storage conditions that could result in the accumulation of SNPs. A study comparing isolates from a single processing facility spanning 12 years in their isolation dates identified one SNP, though there was evidence of plasmid and prophage alterations [12]. It is important to consider that while there was less change in these processing facilities isolates, they were known to be from a related source that, while uncontrolled, may have provided more similar selective pressures than the environments for LS47 and LS114 which were isolated from different sources (clinical vs. environmental) and underwent subsequent laboratory passage.

We also compared the isolates identified as Cluster 1 (S2 Table) using the CFSAN SNP Pipeline with F2365 as the reference genome. This analysis identified 156 SNPs in this cluster relative to F2365; however, while three were completely unique to LS651 with the F2365 nucleotide conserved in the older isolates, an additional 21 SNPs had a point mutation that differed from those found at that site in the older isolates. For example, LS267 encoding a G while LS651 encoded a T and the others had maintained the F2365 residue at a given location. This data supports a remarkable level of relatedness and given the time span involved, could again represent divergence of the LS651 strain from the original population represented by the 1994 isolates.

## Discussion

Serotyping of Lm strains during outbreak investigations is the first step towards subtyping. Although a vast majority of the strains linked to outbreaks are grouped into three serotypes, 1/2a, 1/2b and 4b, occasional deviation from this pattern may provide a very important clue during early phases of outbreak investigations. For example, a listeriosis outbreak in Finland involving butter was caused by serotype 3a strains [36]. We have been using a real-time PCR-based assay developed in our laboratory for serotyping of Lm strains that is based on a previously published PCR protocol for sero-grouping strains [18, 26]. The protocol identifies the major disease causing serotypes and a few other rare serotypes including 1/2c, 3a, 3b, 3c, when coupled with a basic agglutination assay, and is uniquely capable of identifying 4bV isolates. Identification of serotype 4bV among the stone fruit and caramel apple outbreak isolates provided the first clue that these strains could be related. Although 4bV isolates of Lm have been reported from different parts of the world [30], including isolates in Australia [34], these variants are not frequently detected. The unique acquisition of a 6.3 kb DNA fragment in their 4b genome backbone indicate that these strains may have acquired newer traits from other *L*. *monocytogenes* serotypes which could affect their pathophysiology and environmental adaptation[29]. The WGS comparison studies of these 4bV strains performed in this study, based on the serotype information, resulted in the identification of an intriguing link that may indicate the need for further evaluation.

Genomic comparison using WGS data has been a very useful and powerful tool for establishing potential links between clinical, food and environmental isolates of *L*. *monocytogenes*. Finding such links during outbreak investigations could lead to identification of the source of contamination, removal of contaminated foods from circulation thereby saving lives and reducing other burdens associated with such outbreaks. Several methods of varying degree of discriminatory power are currently in use for analysis of WGS data. These bioinformatics tools, however, are resource intensive and can take a long time particularly if one has to compare dozens of isolate genomes. In this communication, we evaluated a relatively easier tool, JSpecies Tetra, to compare genomes of several *L*. *monocytogenes* strains obtained from various listeriosis outbreaks. As a first step towards this goal, we compared the genomes of *L*. *monocytogenes* isolates, which had been already established to be linked with each other by other genomics tools and epidemiological investigation.

The results with the limited numbers of epidemiologically linked outbreak strains clearly showed that JSpecies Tetra analysis could be a useful tool to indicate a genomic relationship. The ready identification of previously known links between *Listeria monocytogenes* isolates, including three 4bV strains from Australia [29], as well as unknown links between *Listeria monocytogenes* isolates, shows that the JSpecies tool may provide a useful initial approach for the rapid assessment of genomic relatedness that can be readily performed on most PCs, making it more user accessible. As a superficial comparison of the three approaches, the JSpecies analysis of the subset in S2 Table took less than 10 minutes while the BLAST-based SNP approach took 16 minutes. The CFSAN SNP Pipeline of this dataset took about 4 hours to run locally, though this analysis can be done much more rapidly on a high-performance computing cluster or with cloud computing. While this time difference is minimal at least for the first two, a key factor to consider is that both SNP analyses required detailed knowledge of bioinformatics tools and the use of a Linux machine, as well as the identification of the core genome. Conversely, the JSpecies tool can be done as both a QA/QC measure verifying that the genomic DNA isn’t contaminated and as a rapid check for relatedness with other strains by any researcher with access to a standard PC, though assembly data can also provide clues on the sequence quality. Furthermore, the easy interpretation of the results would improve the ability of investigators to identify links and communicate them across a wide range of disciplines as well as to individuals with the skills and tools to perform the more rigorous methods to determine the actual degree of relatedness. Use of the JSpecies Tetra analysis tool would improve the quality of genomic DNA sequence being submitted into GenBank and enable the scientist to conduct their own independent detailed analysis or to alert investigators of any noteworthy links for further more detailed analysis.

While evaluating this tool, we identified two cases of genetic relatedness that would have been relevant if the information had been available to investigators during the active response in either the 2011 case or the caramel apple outbreak. In the case of the links between the 2014 isolates, the question remains as to why these highly related strains were found in two unrelated food vehicles, as well as in clinical isolates from the summer of 2014 that have not been linked to a food source [19]. The production facilities for these foods are located about 70–80 miles from each other. It is possible that the strains implicated in these incidents is present natively within the region and may have adapted to be more competitive in this environment with a coincidental increased fitness in survival within packing facilities. Alternatively, it is possible that a cross-contamination event occurred between the facilities. Determination of which of these explanations is more likely would allow better control of future incidents.

The slight divergence between the fruit 4bV isolates suggests the possibility of an environmental niche favorable to the expansion of these strains within the growing region or within the processing facilities. It is interesting that the stone fruit isolates had a higher frequency and specificity of SNPs than the apple isolates suggesting the possibility of a shift selective pressure in the stone fruit environment driving the accumulation of mutations. Given that both foods were fruits, it should be investigated whether this 4bV strain is uniquely adapted to produce or, specifically, fruit contamination and whether there are factors unique to the stone fruits or their processing environment that could explain the greater divergence from the reference strain.

In summary, this evaluation of the JSpecies tool has shown that it can rapidly and easily compare WGS of Lm strains, establish genetic relatedness, especially when aided by a simple PCR based serogrouping, and provide useful information guiding source attribution efforts and improving outbreak response.

## Supporting Information

S1 Table Strains for JSpecies Base Assessment. This table provides the information on the strains, as well as their biosample number, used to assess JSpecies performance.
(XLSX)

S2 Table JSpecies Comparison of 4b and 4bV Isolates. Highly related groups are highlighted with previously known clusters highlighted grey and the new clusters in purple (Cluster 1), green (Cluster 2) and blue (Cluster 3). The blue highlighted text shows the r^2^ values for previously characterized 4bV isolates.
(XLSX)

S3 Table CFSAN Pipeline identified SNPs for Cluster 3. This table lists the SNPs found in the cluster 3 strains relative to LS642 that occurred that were found more frequently in one of the two strain subset. A period (.) indicates the nucleotide was unaltered in the test strain relative to LS642 while a dash (-) indicates a gap. Each SNP was assigned as SF specific, SF biased or apple biased as noted by the position of the plus symbol.
(XLSX)

## References

[1] CDC. Multistate outbreak of listeriosis associated with Jensen Farms cantaloupe—United States, August-September 2011. MMWR Morb Mortal Wkly Rep. 2011;60(39):1357–8. 21976119

[2] CDC. Multistate Outbreak of Listeriosis Linked to Blue Bell Creameries Products (Final Update) 2015 89updated 6/10/2015]. Available: http://www.cdc.gov/listeria/outbreaks/ice-cream-03-15/index.html.

[3] CDC. Multistate Outbreak of Listeriosis Linked to Commercially Produced, Prepackaged Caramel Apples Made from Bidart Bros. Apples (Final Update) 2015 [updated 2/12/2015]. Available: http://www.cdc.gov/listeria/outbreaks/caramel-apples-12-14/index.html.

[4] Ruppitsch W, Pietzka A, Prior K, Bletz S, Fernandez HL, Allerberger F, et al Defining and Evaluating a Core Genome Multilocus Sequence Typing Scheme for Whole-Genome Sequence-Based Typing of Listeria monocytogenes. J Clin Microbiol. 2015;53(9):2869–76. 10.1128/JCM.01193-15 26135865 PMC4540939

[5] FDA. GenomeTrakr Network 2015 [updated 2015]. Available: http://www.fda.gov/Food/FoodScienceResearch/WholeGenomeSequencingProgramWGS/ucm363134.htm.

[6] CDC. Maximizing the potential of real-time whole genome sequence-based Listeria surveillance to solve outbreaks and improve food safety 1908 [updated 8/18/15]. Available: http://www.cdc.gov/amd/project-summaries/listeria.html.

[7] Hoffmann M, Luo Y, Monday SR, Gonzalez-Escalona N, Ottesen AR, Muruvanda T, et al Tracing Origins of the Salmonella Bareilly Strain Causing a Food-borne Outbreak in the United States. J Infect Dis. 2016;213(4):502–8. 10.1093/infdis/jiv297 25995194

[8] Schmid D, Allerberger F, Huhulescu S, Pietzka A, Amar C, Kleta S, et al Whole genome sequencing as a tool to investigate a cluster of seven cases of listeriosis in Austria and Germany, 2011–2013. Clin Microbiol Infect. 2014;20(5):431–6. 10.1111/1469-0691.12638 24698214 PMC4232032

[9] Jackson BR, Salter M, Tarr C, Conrad A, Harvey E, Steinbock L, et al Notes from the field: listeriosis associated with stone fruit—United States, 2014. MMWR Morb Mortal Wkly Rep. 2015;64(10):282–3. 25789745 PMC4584806

[10] CDC. Multistate Outbreak of Listeriosis Linked to Roos Foods Dairy Products (Final Update) 2014 [updated 4/18/2014]. Available: http://www.cdc.gov/listeria/outbreaks/cheese-02-14/.

[11] Mohammed M, Delappe N, O'Connor J, Mc KP, Garvey P, Cormican M. Whole genome sequencing provides an unambiguous link between Salmonella Dublin outbreak strain and a historical isolate. Epidemiol Infect. 2016;144(3):576–81. 10.1017/S0950268815001636 26165314

[12] Orsi RH, Borowsky ML, Lauer P, Young SK, Nusbaum C, Galagan JE, et al Short-term genome evolution of Listeria monocytogenes in a non-controlled environment. BMC Genomics. 2008;9:539 10.1186/1471-2164-9-539 19014550 PMC2642827

[13] Gardner SN, Hall BG. When whole-genome alignments just won't work: kSNP v2 software for alignment-free SNP discovery and phylogenetics of hundreds of microbial genomes. PLoS One. 2013;8(12):e81760 10.1371/journal.pone.0081760 24349125 PMC3857212

[14] Pightling AW, Petronella N, Pagotto F. Choice of reference sequence and assembler for alignment of Listeria monocytogenes short-read sequence data greatly influences rates of error in SNP analyses. PLoS One. 2014;9(8):e104579 10.1371/journal.pone.0104579 25144537 PMC4140716

[15] Pightling AW, Petronella N, Pagotto F. Choice of reference-guided sequence assembler and SNP caller for analysis of Listeria monocytogenes short-read sequence data greatly influences rates of error. BMC Res Notes. 2015;8:748 10.1186/s13104-015-1689-4 26643440 PMC4672502

[16] Pettengill JB, Luo Y, Davis S, Chen Y, Gonzalez-Escalona N, Ottesen A, et al An evaluation of alternative methods for constructing phylogenies from whole genome sequence data: a case study with Salmonella. PeerJ. 2014;2:e620 10.7717/peerj.620 25332847 PMC4201946

[17] Richter M, Rossello-Mora R. Shifting the genomic gold standard for the prokaryotic species definition. Proc Natl Acad Sci U S A. 2009;106(45):19126–31. 10.1073/pnas.0906412106 19855009 PMC2776425

[18] Burall LS, Simpson AC, Datta AR. Evaluation of a serotyping scheme using a combination of an antibody-based serogrouping method and a multiplex PCR assay for identifying the major serotypes of Listeria monocytogenes. J Food Prot. 2011;74(3):403–9. 10.4315/0362-028X.JFP-10-355 21375876

[19] CDC. Listeriosis Associated with Stone Fruit—United States, 2014. MMWR Morb Mortal Wkly Rep. 2015;64(10):282–3. 25789745 PMC4584806

[20] Laksanalamai P, Jackson SA, Mammel MK, Datta AR. High density microarray analysis reveals new insights into genetic footprints of Listeria monocytogenes strains involved in listeriosis outbreaks. PLoS One. 2012;7(3):e32896 10.1371/journal.pone.0032896 22457724 PMC3310058

[21] Hain T, Ghai R, Billion A, Kuenne CT, Steinweg C, Izar B, et al Comparative genomics and transcriptomics of lineages I, II, and III strains of Listeria monocytogenes. BMC Genomics. 2012;13:144 10.1186/1471-2164-13-144 22530965 PMC3464598

[22] Nelson KE, Fouts DE, Mongodin EF, Ravel J, DeBoy RT, Kolonay JF, et al Whole genome comparisons of serotype 4b and 1/2a strains of the food-borne pathogen Listeria monocytogenes reveal new insights into the core genome components of this species. Nucleic Acids Res. 2004;32(8):2386–95. 15115801 10.1093/nar/gkh562 PMC419451

[23] den Bakker HC, Desjardins CA, Griggs AD, Peters JE, Zeng Q, Young SK, et al Evolutionary dynamics of the accessory genome of Listeria monocytogenes. PLoS One. 2013;8(6):e67511 10.1371/journal.pone.0067511 23825666 PMC3692452

[24] Chen Y, Strain EA, Allard M, Brown EW. Genome sequences of Listeria monocytogenes strains J1816 and J1-220, associated with human outbreaks. J Bacteriol. 2011;193(13):3424–5. 10.1128/JB.05048-11 21551300 PMC3133268

[25] Briers Y, Klumpp J, Schuppler M, Loessner MJ. Genome sequence of Listeria monocytogenes Scott A, a clinical isolate from a food-borne listeriosis outbreak. J Bacteriol. 2011;193(16):4284–5. 10.1128/JB.05328-11 21685277 PMC3147710

[26] Doumith M, Buchrieser C, Glaser P, Jacquet C, Martin P. Differentiation of the major Listeria monocytogenes serovars by multiplex PCR. J Clin Microbiol. 2004;42(8):3819–22. 15297538 10.1128/JCM.42.8.3819-3822.2004 PMC497638

[27] Tamura K, Stecher G, Peterson D, Filipski A, Kumar S. MEGA6: Molecular Evolutionary Genetics Analysis version 6.0. Mol Biol Evol. 2013;30(12):2725–9. 10.1093/molbev/mst197 24132122 PMC3840312

[28] Saitou N, Nei M. The neighbor-joining method: a new method for reconstructing phylogenetic trees. Mol Biol Evol. 1987;4(4):406–25. 3447015 10.1093/oxfordjournals.molbev.a040454

[29] Laksanalamai P, Huang B, Sabo J, Burall LS, Zhao S, Bates J, et al Genomic characterization of novel Listeria monocytogenes serotype 4b variant strains. PLoS One. 2014;9(2):e89024 10.1371/journal.pone.0089024 24586485 PMC3929640

[30] Leclercq A, Chenal-Francisque V, Dieye H, Cantinelli T, Drali R, Brisse S, et al Characterization of the novel Listeria monocytogenes PCR serogrouping profile IVb-v1. Int J Food Microbiol. 2011;147(1):74–7. 10.1016/j.ijfoodmicro.2011.03.010 21470706

[31] Lee S, Ward TJ, Graves LM, Wolf LA, Sperry K, Siletzky RM, et al Atypical Listeria monocytogenes serotype 4b strains harboring a lineage II-specific gene cassette. Appl Environ Microbiol. 2012;78(3):660–7. 10.1128/AEM.06378-11 22138999 PMC3264116

[32] Felsenstein J. Confidence limits on phylogenies: An approach using the bootstrap. Evolution. 1985;39:783–91. 28561359 10.1111/j.1558-5646.1985.tb00420.x

[33] Nei M, Kumar S. Molecular Evolution and Phylogenetics. New York: Oxfird University Press; 2000 2000.

[34] Huang B, Fang N, Dimovski K, Wang X, Hogg G, Bates J. Observation of a new pattern in serogroup-related PCR typing of Listeria monocytogenes 4b isolates. J Clin Microbiol. 2011;49(1):426–9. 10.1128/JCM.01207-10 21048013 PMC3020444

[35] Linnan MJ, Mascola L, Lou XD, Goulet V, May S, Salminen C, et al Epidemic listeriosis associated with Mexican-style cheese. N Engl J Med. 1988;319(13):823–8. 3137471 10.1056/NEJM198809293191303

[36] Lyytikainen O, Autio T, Maijala R, Ruutu P, Honkanen-Buzalski T, Miettinen M, et al An outbreak of Listeria monocytogenes serotype 3a infections from butter in Finland. J Infect Dis. 2000;181(5):1838–41. 10823797 10.1086/315453

